# Brain-wide dendrites in a near-optimal performance of dynamic range and information transmission

**DOI:** 10.1038/s41598-023-34454-8

**Published:** 2023-05-09

**Authors:** Congping Lin, Fan Xu, Yiwei Zhang

**Affiliations:** 1 School of Mathematics and Statistics and Center for Mathematical Sciences, Huazhong University of Science and Technology, Wuhan, China; 2 Hubei Key Lab of Engineering Modeling and Scientific Computing, Huazhong University of Science and Technology, Wuhan, China; 3 Department of Mathematics, Southern University of Science and Technology, Shenzhen, Guangdong China

**Keywords:** Complex networks, Network models

## Abstract

Dendrites receive and process signals from other neurons. The range of signal intensities that can be robustly distinguished by dendrites is quantified by the dynamic range. We investigate the dynamic range and information transmission efficiency of dendrites in relation to dendritic morphology. We model dendrites in a neuron as multiple excitable binary trees connected to the soma where each node in a tree can be excited by external stimulus or by receiving signals transmitted from adjacent excited nodes. It has been known that larger dendritic trees have a higher dynamic range. We show that for dendritic tress of the same number of nodes, the dynamic range increases with the number of somatic branches and decreases with the asymmetry of dendrites, and the information transmission is more efficient for dendrites with more somatic branches. Moreover, our simulated data suggest that there is an exponential association (decay resp.) of overall relative energy consumption (dynamic range resp.) in relation to the number of somatic branches. This indicates that further increasing the number of somatic branches (e.g. beyond 10 somatic branches) has limited ability to improve the transmission efficiency. With brain-wide neuron digital reconstructions of the pyramidal cells, 90% of neurons have no more than 10 dendrites. These suggest that actual brain-wide dendritic morphology is near optimal in terms of both dynamic range and information transmission.

## Introduction

Dendritic trees of neurons receive, process and transfer information from other neurons to the soma. Computational models have been developed and proposed to describe complex dendritical structures^1–5^. Optimization models have also been developed to understand the intrinsic mechanisms underling branching^6–8^. It has been suggested that dendrites grow to fill a target space in an optimal manner, using the least amount of wiring to reach all synaptic contacts. Moreover, besides intrinsic mechanisms, branching morphogenesis may also be controlled externally^9–11^.

The complex morphological structure of dendritic trees is likely associated with the function^12,13^. Dendritic branches may support clustered inputs, connection specificity, and dendritic computation^14^. Dendrites have been shown to exhibit excitability through the expression of a variety of voltage-gated ion channels, and are suggested to associate with computational functions such as the learning capacity^15^. Moreover, considering dendrites as spatially extended excitable media, enhancement of dynamic range is suggested to be the main functional role of active dendritic conductances^16^.

The dynamic range is a quantity that measures the range of afferent rate that a neuron recognizes, discarding stimuli which are either too weak to be distinguished of the system or too close to saturation^16^. A number of works have been done to study optimal dynamic range in neural networks^17–22^. Particularly for dendrites (often modelled as composition of binary trees connecting to the soma), it has been suggested that larger binary trees have larger dynamic range and that blocking of active dendritic branchlets in binary trees would lead to a decrease in dynamic range^16,23^ using both mean-field approximation and stochastic simulations. More recently, using digital reconstructions of neurons from the NeuroMorpho database, it has been suggested that the location of the soma and the number of somatic branches are key topological factors in determining both the neuron’s dynamic range as well as its energy consumption^24^.

In this manuscript we extend studies of dynamic range by including asymmetry and multiple branches for dendrites connected to the soma. To exclude the effect of node number, we consider dendrites with the same number of nodes in modelling. We observe that double-sigmoid response to external stimuli is more visible when dendrites have more somatic branches, and thus we adapt the definition of dynamic range. Our simulated data suggest that the dynamic range increases with the number of somatic branches exponentially to a plateau, and the overall energy consumption decays exponentially with the number of somatic branches. Beyond an intermediate number of somatic branches, further increasing the number of branches has limited ability to improve dynamic range or information transmission efficiency. Together with digital reconstructions of brain-wide neurons, our simulated results suggests that actual dendritic morphology appears to be near optimal with an intermediate number of somatic branches, in terms of both dynamic range and information transmission.

## Results

### Double-sigmoid response and dynamic range

**Figure 1:**
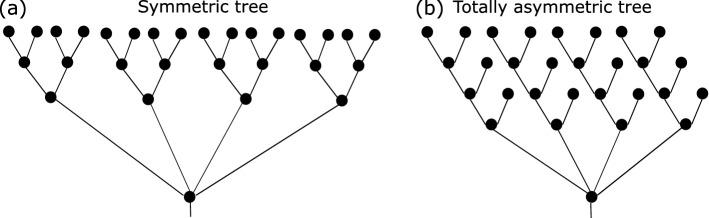
Illustration of “symmetric” and “totally asymmetric” trees with four branches (i.e. $\#B=4$) connected to the soma.

In this section, we study the dynamic range of dendrites and its relation to dendritic topology. We characterize the dendritic topology by its number of somatic branches (denoted as $\#B$) and tree asymmetry (denoted as *A* and given in Eq. (1) in “Methods”). Figure 1 shows two extreme (“symmetric” and “totally asymmetric”) examples of dendritic trees.

#### Response function and dynamic range

**Figure 2:**
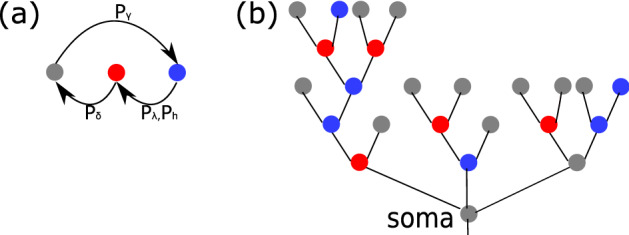
(**a**) Three potential states of each node in a dendrite: resting (blue), excited (red) and refractory (grey); a node in resting state can be excited by an external stimulus with probability $P_h$ or by an excited neighbor with probability $P_\lambda$, an excited node changes to a refractory state with probability $P_\delta$ and a refractory node changes to rest with probability $P_{\gamma }$, after a single time step. (**b**) An illustration of a dendrite with three branches; the state for each node is colored according to (**a**).

To account for the active nature of dendrites, we model each node in dendrites as a simple discrete excitable element^16,17^, which can be either in a resting, refractory or excited state. A resting node can be excited by external stimulus with probability $P_h$, i.e. receiving signals from other synapses in a Poisson process with a rate *h*, or by receiving signals transmitted by adjacent excited nodes with probability $P_{\lambda }$; see Fig. 2 and “Methods” for model details.

**Figure 3:**
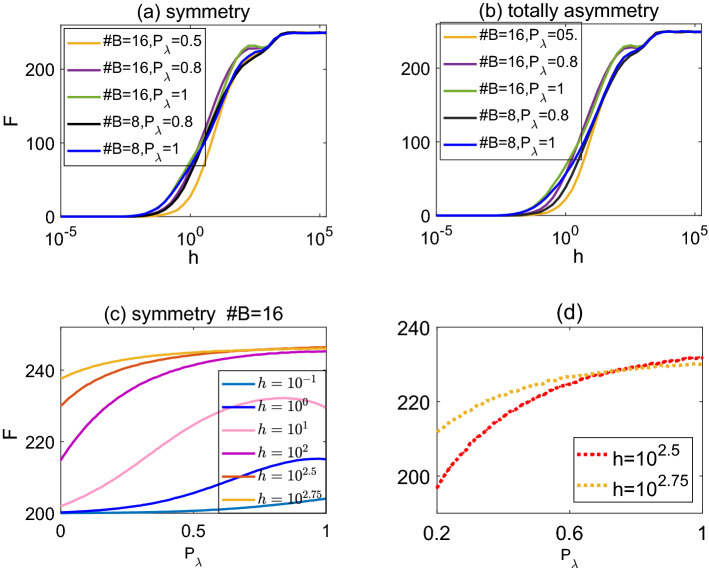
(**a**, **b**) Double sigmoid response curves *F*(*h*) at various interaction probability $P_\lambda$ for symmetric (**a**) and totally asymmetric (**b**) dendrites with different number of somatic branches (indicated by $\#B$). Note that some response curves *F*(*h*) decrease before reaching saturation. (**c**) The response *F* in relation to $P_\lambda$ at various external stimulation rate *h* for a symmetric dendrite with 16 branches. The zoomed curves for $h=10^{2.5}$ and $h=10^{2.75}$ are shown in panel (**d**) where two response curves intersect at around $P_\lambda \approx 0.7$.

The firing activity (also referred to as response function) *F* is quantified as the average number of excitations produced at the soma averaged over a large time window. The dependence of response function *F*(*h*) on the stimulus rate *h* has a saturation aspect and Hill functions are widely employed to study the response function. However, double-sigmoid behavior was observed in experimental data^25^. Such double-sigmoid has been successfully modelled in virtual symmetric trees^16^ in a single binary tree. Considering neurons usually have multiple dendrites, we model dendrites in a neuron as a tree with multiple binary subtrees connecting to the root (see Fig. 2 as an example of a tree with 3 branches). We find that in our active dendrite model, double-sigmoid response curves commonly appear in dendrites with multiple branches when interaction probability $P_{\lambda }$ is high; in particular, the double sigmoid behavior is more visible for dendritic trees with more somatic branches at higher interaction probability, as seen in Fig. 3a,b.

Moreover, Fig. 3 shows that for a high spiking interaction $P_{\lambda }=1$ and a large number of somatic branches (e.g. with a branching number $\#B=16$), the response curve *F*(*h*) decreases within a certain range of stimulus rate *h* in the first sigmoid. This means increasing the stimulating rate in a certain range, reduces the average firing rate. To further study this short drop in response, we plot the response activity *F* as a function of spiking interaction probability $P_\lambda$ (see Fig. 3c,d). Indeed, for some range of *h*, curves $F(P_\lambda )$ crossover and beyond the crossing point, higher *h* gives lower response activity *F*. Moreover, we observe for some intermediate *h* that the activity $F(P_\lambda )$ is maximized at some intermediate interaction probability $P_{\lambda }$ (see Fig. 3c); this is referred to as screening resonance in^16^. For low enough $P_\lambda$, excitations created in distal nodes may not arrive at the soma due to propagation failure, whereas for too strong interaction, backward propagation of activity effectively can block forward propagation of incoming signals.

**Figure 4:**
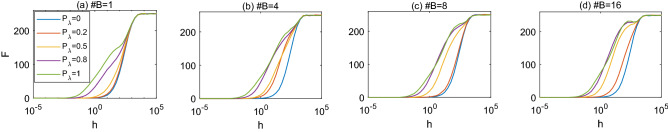
Response curves *F*(*h*) at various interaction probability $P_\lambda =0,0.2,0.5,0.8,1$ for symmetric trees with somatic branching number $\#B=1,4,8,16$ as indicated in each panel. Double sigmoid shape is more visible for higher $P_\lambda$ and higher number of somatic branches.

It has been suggested that double sigmoid behavior is related to two different modes of activation at the soma^16^: the first mode is related to the complicated interactions between spikes in the tree, and the second mode is the direct excitation due to the stimulation rate *h*. Such two modes also appears for trees with multiple branches. Indeed, for trees with no interactions between nodes (i.e., $P_\lambda =0$), the double sigmoidal behaviour disappears and only the second sigmoid remains (at large *h*); see Fig. 4 for both single-branch and multiple-branch dendrites. With a large interaction strength between spikes (i.e., large $P_\lambda$), double sigmoidal behaviour occurs; see Fig. 4 ($P_\lambda \ge 0.8$). Interestingly, we observe that with a large number of somatic branches, the first sigmoid appears even at low $P_{\lambda }$ (e.g., dendrites with 16 branches at $P_\lambda =0.5$ as shown in Fig. 3). This might be that when the soma is connected to multiple branches, it can receive signals from all these branches, which leads to somatic excitability saturation at a low interaction probability $P_\lambda$.

**Figure 5:**
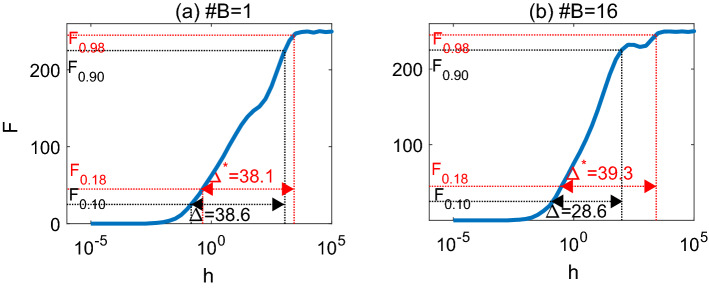
(**a**) The response curve *F*(*h*) for a single-branch symmetric dendrite with 256 nodes. *F*(*h*) exhibits a single sigmoid shape, and the (revised resp.) dynamic range is calculated as $\Delta =38.6$ (and $\Delta ^{*}=38.1$ resp.). (**b**) The response curve *F*(*h*) for a symmetric dendrite with 16 branches. The corresponding response curve *F*(*h*) exhibits double sigmoid shape and the (revised resp.) dynamic range is calculated as $\Delta =28.6$ (and $\Delta ^{*}=39.3$ resp.). Parameter $P_{\lambda }=1$ is used in both panels.

The response *F*(*h*) which may exhibit double sigmoid behaviour and may decrease within a certain range of stimulation rate *h*, we thus use the calculation of revised dynamic range $\Delta ^*$ (see Eq. (3) in “Methods” for details) for trees with various number of branches. This calculation of the revised dynamic range gives similar values to the previous definition $\Delta$ for single-branch trees; see Fig. 5a for an example. It has been shown that dynamic range of symmetric binary trees increase with node number^16^. This property also holds with the revised dynamic range $\Delta ^*$. In the following, we refer to $\Delta ^*$ as dynamic range without ambiguity.

#### Topological effects on dynamic range

**Figure 6:**
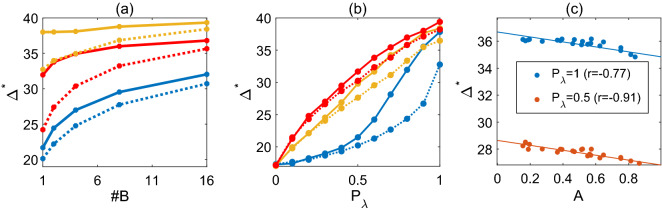
(**a**) The dynamic range of symmetric (solid lines) and totally asymmetric (dashed lines) dendrites of $N=256$ nodes with various number of somatic branches at different $P_\lambda$ values; from top to bottom lines $P_\lambda =1$ (orange), 0.8 (red), 0.5 (blue) respectively. (**b**) The dynamic range of symmetric (solid) and totally asymmetric (dashed) dendrites of $N=256$ nodes with 1(blue), 8(orange), or 16(red) branches at various $P_\lambda$. Note that symmetric dendrites have higher dynamic range than totally asymmetric dendrites when $P_\lambda$ is high. (**c**) The dynamic range $\Delta ^*$ of dendrites with $N=128$ nodes and 7 branches for various tree asymmetry *A*. Note that $\Delta ^*$ strongly correlates with tree asymmetry *A*; the person correlation coefficient *r* is indicated.

Neurons have various number of somatic branches. We first study the influence of branching number on the dynamic range. It is clear that for symmetric trees, the more branches the larger number of nodes, and a larger dynamic range would be expected. To exclude the effect of node number, we fixed a total number of nodes $N=256$ in dendrites and show in Fig. 6a that for both “symmetric” and “totally asymmetric” dendrites of the same number of nodes, the dynamic range increases with number of branches as well as the interaction probability $P_\lambda$. This is consistent with published results^24^ for neurons from the online repository NeuroMorpho database, though total number of nodes may vary among neurons there.

Actual dendrites are usually neither symmetry nor totally asymmetric. We next consider tree asymmetry and its association with dynamic range. We observe in Fig. 6a,b that totally asymmetric dendrites have no larger dynamic range than symmetric dendrites and the difference of dynamic range between symmetric and totally asymmetric dendrites magnifies with less branches and higher $P_\lambda$. In particular, we show in Fig. 6b that tree asymmetry has little effect on dynamic range for a low interaction probability $P_{\lambda }$, whereas for high $P_{\lambda }$, symmetric trees have clear higher dynamic range than totally asymmetric trees. This can be explained that at a low interaction probability, the somatic excitation is mainly due to the stimulation rate *h*, and thus tree topological difference makes little difference on dynamic range. For trees with equal number of nodes, compared to totally asymmetric trees, symmetric trees have less generations and thus overall non-soma nodes take less time to activate the soma in particular when the interaction $P_{\lambda }$ comes into play for the response at the soma.

To further investigate the asymmetry on dynamic range, we generate random trees with a fixed number of somatic branches ($\#B=7$, different subtree may exhibit different topology) and a fixed number of tree nodes $N=128$, and calculate their tree asymmetry *A* as well as the corresponding dynamic range $\Delta ^*$. We shown in Fig. 6c that the dynamic range $\Delta ^{*}$ strongly correlates with tree asymmetry *A*, and generally, the dynamic range decreases linearly with tree asymmetry for high interaction probability $P_{\lambda }$.

### Energy consumption and transmission efficiency

**Figure 7:**
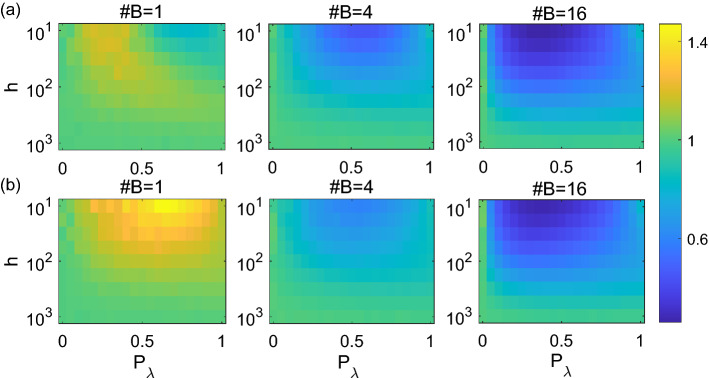
Illustration of the relative energy consumption *E* in relation to interaction probability $P_\lambda$ and stimulation rate *h* in an intermediate range $h\in [10^1,10^3]$ for both symmetric (**a**) and totally asymmetric (**b**) dendrites; the number of somatic branches in dendrites is indicated in each panel. Limits of the colorbar are set to be the same among the panels for visitation effect.

Dendrites generating spikes for enhancement of activity and the spiking interactions require energy. We compute relative energy consumption *E* (see “Methods” for details) which quantifies how many times on average dendritic compartments activate for each somatic spike as in^24^. A higher energy consumption *E* indicates less efficiency in spiking transmission. Clearly, for $P_\lambda =0$ or sufficiently large *h*, one has $E=1$. For small *h*, large fluctuations are expected, thus we consider intermediate stimulation rates *h* and show the computed relative energy consumption *E* in Fig. 7. For single-branch dendrites, the relative energy consumption is maximized at intermediate $P_\lambda$ and relatively small *h* (with maximized $E>1$), whereas for dendrites with multiple branches, the relative energy consumption is lower at intermediate $P_\lambda$ and small *h* (with $E\lesssim 1$), as seen from Fig. 7. This is probably due to that for single-branch dendrites, the soma has its unique neighbor to interact with. Note that the overall energy consumption is higher for single-branch dendrites than those with multiple branches. With more branches, it is easier for the first few nodes (which are close to soma) to activate the soma. These results suggest that more branches in dendrites lead to an increase in the transmission efficiency to the soma. Moreover, with the same number of nodes, totally asymmetric single-branch dendrites overall cost slightly more energy than symmetric dendrites, as seen from Fig. 7.

**Figure 8:**
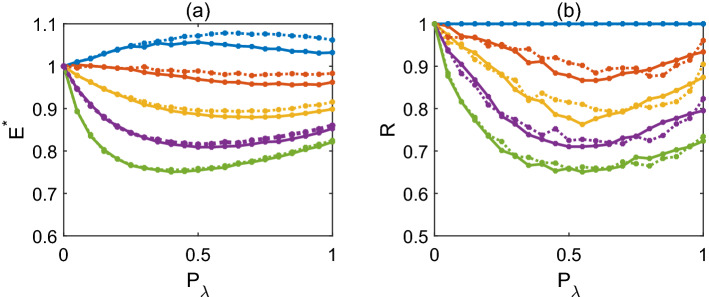
(**a**) The average of relative energy consumption $E^*$ in relation to the interaction probability $P_\lambda$ for different somatic branching number of symmetric (solid curves) and totally asymmetric (dotted curves) dendrites. (**b**) That the dynamic range ratio *R* in relation to the interaction probability $P_\lambda$ for symmetric (solid curves) and totally asymmetric (dotted curves) dendrites. The number of somatic branches in dendrites is 1 (blue), 2 (orange), 4 (yellow), 8 (purple) or 16 (green) from top to bottom in each panel.

To quantify the overall energy consumption, we take the average of relative energy consumption $E^*$ over a range of external stimulation rate *h* (see “Methods” for details). As expected, we see in Fig. 8a that symmetric dendrites have slight lower $E^*$ than totally asymmetric dendrites with a small number of somatic branches in particular for single-branch dendrites when $P_\lambda$ is high. Moreover, for single-branch dendrites, the average of relative energy consumption $E^*\ge 1$ whereas for multiple-branch dendrites $E^*\le 1$ in particular when $P_\lambda$ is high. We also see from Fig. 8a that the average of relative energy consumption $E^*$ decreases with the number of somatic branches at various interaction probability $P_\lambda$.

Besides the average of relative energy consumption $E^*$, we also calculate dynamic range ratio *R* (see Eq. (6) in “Methods” for details) by the ratio of dynamic range from individual subtrees to the whole multiple-branch dendrite as another indicator for information transmission efficiency. A low ratio *R* indicates an effectively large magnification of the stimulus range of afferent rate that dendrites recognize due to multiple branches, meaning a large magnification of dynamic range when individual subtrees connected as a whole dendritic tree. Clearly, one has $R=1$ for single-branch dendrites, and $R\le 1$ for dendrites with multiple branches. Similar to the average of relative energy consumption $E^*$, we show in Fig. 8b that the ratio *R* is lower for dendrites with more branches, meaning a larger magnification effect on dynamic range. Also note that the dynamic range ratio *R* as well as the average of relative energy consumption $E^*$ for dendrites with multiple branches are lower for intermediate $P_\lambda$; this indicates that generally intermediate interaction probability $P_\lambda$ gives more efficiency for dendrite information transmission.

### Dynamic range from real brain-wide dendrites

**Figure 9:**
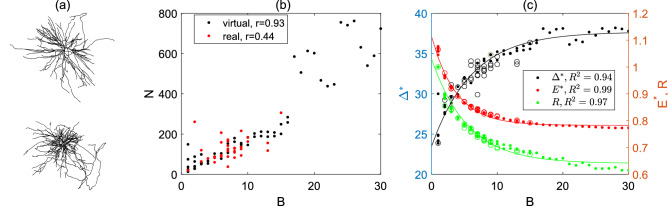
(**a**) Two examples of brain-wide neuron digital reconstructions of Pyramidal cells; the brain is from C57BL/6J mouse line^4,26^. (**b**) The correlation of node number and number of branches connected to the soma in dendrites for both virtually generated random dendrites (black) and actual brain-wide dendrites (red); Pearson correlation value *r* is indicated and $p<0.05$, suggesting a strong positive correlation between the number of nodes and the number of branches. (**c**) The dynamic range $\Delta ^*$, average of relative energy consumption $E^*$ and dynamic range ratio *R* in relation to the number of branches in virtual random constructed dendrites (dots) as well as actual brain-wide dendrites (circles). Here we use $P_\lambda =0.8$ in computation. Goodness of fit $R^2$ is indicated for each curve.

In this section we study real brain-wide dendritic topology and its association with dynamic range and information transmission characterized via the average of relative energy consumption and dynamic range ratio. The real dendrites we use are taken from published brain-wide neuron digital reconstructions of the Pyramidal cells in a whole C57BL/6J mouse brain from^4,26^; two brain-wide dendrite digital reconstructions are shown in Fig. 9a. Note that the dynamic range is maximized at $P_{\lambda }=1$ whereas the information transmission is optimal at an intermediate $P_{\lambda }$ for multi-branch dendrites. Considering both the dynamic range and energy consumption in Figs. 6 and 8, we choose $P_{\lambda }=0.8$ (at which both the dynamic range and energy consumption are near the optimal) in this section to investigate real brain-wild dendrites; results are similar for other values of $P_{\lambda }$ (data not shown).

For dendrites in actual neurons, they may have various asymmetry and various number of nodes. To take account of various dendrite topology, we generate 55 virtual random dendrites of various asymmetry, and various number of nodes or branches. Note that the number of nodes is positively correlated with the number of branches for both virtual and actual dendrites; see Fig. 9b; moreover, Fig. 9b also show that the virtual dendrites we generated have similar node number as actual dendrites if they have the same number of somatic branches.

We show in Fig. 9c that as expected among the virtual random dendrites as well as actual brain-wide dendrites, dynamic range $\Delta ^*$ increases with the number of somatic branches while the average of relative energy consumption $E^*$ and dynamic range ratio *R* decrease with the number of somatic branches. With a large number of somatic branches we considered in simulations, Fig. 9c show that these data are well fit to an exponential decay/association: the dynamic range $\Delta ^*$ increases to almost saturation with the number of branches, and the relative energy consumption $E^*$ and dynamic range ratio *R* decrease to almost flat with the number of branches. These indicate that with further increase of the number of branches, the improvement for dynamic range and information transmission efficiency is limited. However, increasing the number of branches indicates an increase of number of nodes (bifurcations) generally, which might require extra materials for neurons.

The actual brain-wide neurons we use from^4,26^ have on average $\#B=7.2\pm 0.53 (n=33)$ dendrites and 90% of these brain-wide neurons have no more than 10 branches. We show in Fig. 9c that the dynamic range, average of relative energy consumption as well as dynamic range ratio of real dendrites are close to those generated from virtual dendrites with the same number of branches and moreover majority of them are near the saturation or plateau in the exponential fit of simulated data. We thus consider real dendrites may take a near-optimal performance in dynamic range and computational efficiency.

## Conclusion and discussion

In this manuscript we employ a simplified excitable model for dendritic dynamics to study multi-compartment dendritic morphology in relation to its computation and information transformation. Although such a moodeling approach lacks biological realism, it has already been shown to be helpful to investigate neuron function such as the enhancement of dynamic range^16^ as well as functional effects of aging and neurodegeneration at the neuronal level^29^, and is a step forward towards more realistic modeling of dendritic dynamics with spiking interaction under natural circumstances^16^.

We study effects of dendrite morphology, in particular the number of somatic branches and dendritic asymmetry, on dynamic range and information transmission. We extend the notion of asymmetry from binary trees to dendrites with multiple binary trees and revise the definition of dynamic range to take account of the double sigmoid behaviour and possible short drop of the firing activity. We show that generally dendritic trees with higher symmetry have higher dynamic range and more efficient in information transmission, though the effect of asymmetry is subtle compared to the influence of the somatic branching number.

Our data suggest that the dynamic range increases in an exponential association manner with the number of somatic branches, and the average of relative energy consumption as well as the dynamic range ratio decay exponentially with the number of somatic branches. This offers a predictor on the dendritic dynamic range and energy consumption; though the exact values may vary based on model parameters, the exponential pattern would be robust.

Furthermore, such an exponential pattern suggests that when the number of somatic branches $\#B$ is sufficiently large, it has only subtle effects on the dynamic range and the average of relative energy consumption. For instance, if the number of somatic branches is increased by two times from $\#B=10$ to $\#B=30$, then the corresponding dynamic range only increases by $\sim 8\%$, the dynamic range ratio only reduces by $\sim 10\%$, and the average of relative energy consumption only reduces by $\sim 5\%$. Among brain-wide neuron digital reconstructions of the Pyramidal cells, the median number of somatic branches is 7, and $90\%$ neurons have no more than 10 somatic branches. The majority of these brain-wide dendrites have an intermediate number of somatic branches, reaching near-optimal performance. Taken together, these suggest that actual dendrite morphology is likely to be in a near-optimal arrangement for both dynamic range and information transmission. Such intermediate number of somatic branches can also be understood by optimal rewiring between given branching points^6,7^; a large number of somatic branches would result in a large path length in dendrites, and optimal wiring between branching points would result in an intermediate number of somatic branches.

Our results also provide an interpretation for the reduction of dynamic range during dendritic pruning in aging and neurodegeneration as reported in^29^. During dendritic pruning, the number of nodes as well the number of somatic branches decrease, and reduction of corresponding dynamic range can be expected, as seen in Fig. 9c. Moreover, a rapid drop on dynamic range for young neurons (with multiple branches) when pruning to a single branch was observed in^29^. This can be explained from the exponential association of the dynamic range, as seen in Fig. 9c; in particular, one can see a much larger change in the dynamic range between dendrites with 1 and 2 branches, compared to that when dendrites have a large number of somatic branches.

## Methods

### Tree asymmetry

For a neuron of *K* dendrites, where each dendrite is modelled as a binary subtree of $N_k$ nodes excluding the soma (i.e., the root), we define the asymmetry of dendrites as below. For a single binary subtree, we calculate its local partition asymmetry $P(r_{j},s_{j})=\frac{\left| r_{j}-s_{j} \right| }{\left| r_{j}+s_{j}-2 \right| }$ following from^4,27^ for all non-terminal nodes; here $r_{j}$ and $s_{j}$ respectively represent the number of terminals in the two branches connected by a non-terminal node *j* and *P*(1, 1) is assigned to 0. We define the asymmetry of a single dendritic branch (e.g., *k*-th dendrite) as

$$\begin{aligned} A_k=\frac{C+\sum _{j}P(r_{j},s_{j})}{n_k}, \end{aligned}$$

here $n_k$ is the number of non-terminals in the *k*-th dendrite. If $C=0$, then this definition of asymmetry of single dendrites is equivalent to the definition given in^4,27^. For binary trees with sufficiently large number of nodes (i.e., sufficiently large $n_k$), if all local partitions $P=1$, then $A_k$ is close to 1, and if all local partitions $P=0$, then $A_k$ is close to 0. Thus $A_k\in (0,1)$. We refer to trees with all local partition $P=1$ ($P=0$ resp.) as “symmetric” (“totally asymmetric” resp.) trees. The asymmetry difference between two “symmetric” subtrees reads as $C|n_1-n_2|/(n_1n_2)$ where $n_{1,2}$ are the number of non-terminal nodes in two subtrees respectively. Similarly, the asymmetry difference of two “totally asymmetric” subtrees reads as $(1-C)|n_1-n_2|/(n_1n_2)$. To consider variations of asymmetry between “symmetric” (or “totally asymmetric”) subtrees with different number of nodes, we set $C=1/2$. We then quantify the asymmetry of dendrites by the weighted average of each dendritic asymmetry as

1 $$\begin{aligned} A=\frac{\sum _{k=1}^{K}N_k\cdot A_k}{\sum _{k=1}^{K} N_k}, \end{aligned}$$

where $N_k$ is the number of nodes in each subtree excluding soma. We refer to dendrites with all totally asymmetric subtrees of equally the same size as “totally asymmetric” dendrites and dendrites with all symmetric subtrees of equally the same size as “symmetric” dendrites. Figure 1 illustrate examples of totally asymmetric and symmetric dendrites.

### Modeling active dendrites

We model dendrites of a neuron as a tree with several branches of binary subtrees representing individual dendrites connecting to the soma in a neuron; see Fig. 2 as an example of a dendritic tree with 3 branches. Nodes in a tree represents a junction or an end of the dendrites. To model the active nature of dendrites, each node can be in one of the three states: resting, excited and refractory at time *t*^17,28^. In one time step *dt*, transitions of node state occur according to below (Fig. 2):

- a node in a resting state can be excited by two ways: one is by external stimulus, i.e. receiving signals from other synapses in a Poisson process with a rate *h* and we model this with a probability $P_{h}=1-\exp (-h\cdot dt)$; the other is by receiving signals transmitted by adjacent excited nodes with probability $P_{\lambda }$;
- a node in an excited state will become refractory with probability $P_{\delta }$;
- a refractory node will change to rest with probability $P_{\gamma }$.

In this manuscript, we use $dt=1$ and simulate for at least $10^4$ steps. We fix $P_{\delta }=1$ and $P_{\gamma }=0.5$ as used in^16^ unless otherwise stated for simplicity.

### Dynamic range

We calculate the somatic activity (response function) *F* as the number of excitations produced at the soma, averaged over $10^4$ time steps and 10 realizations unless otherwise stated. Based on response function *F*(*h*) in relation to the external stimulating rate *h*, the dynamic range was previously defined as^16^

2 $$\begin{aligned} \Delta =10\log _{10}\left(\frac{h_{0.90}}{h_{0.10}}\right). \end{aligned}$$

Here $h_x$ represents the stimulating rate uniquely determined from $F(h_x)=F_x$ where $F_x=F_0+x(F_{\max }-F_0), x\in [0,1]$, assuming *F*(*h*) is monotonically increasing with *h*^17^. Figure 5a shows an illustration of the calculation on dynamic range $\Delta$. However, we find that for a tree with a large number of branches (such as 16 branches in Fig. 5b), double sigmoid occurs in the response curve *F*(*h*). In particular for the response curve in Fig. 5b we observe *F* decreases in a certain range of *h* before it reaches saturation. Such double-Sigmoid behavior appears for various response functions *F*(*h*) (Fig. 3), and we find that the second inflection point occurs near $F_{0.98}$. To be consistent with the span between $F_{0.90}$ and $F_{0.10}$ used in Eq. (2), we revise the calculation of dynamic range using $F_{0.18}$ and $F_{0.98}$ as

3 $$\begin{aligned} \Delta ^{*}=10\log _{10}\left(\frac{h_{0.98}}{h_{0.18}}\right), \end{aligned}$$

and refer to it as *revised dynamic range*. As seen in Fig. 5a, this revised dynamic range $\Delta ^*$ give similar values as previous definition $\Delta$ for single sigmoid response functions *F*(*h*).

### Information transmission efficiency

The relative energy consumption as described in^24,29^ quantifies how active the whole dendritic tree is compared to the soma, that is, how many times, on average, dendritic compartments activate for each somatic spike. Explicitly, for a dentritic tree with *N* nodes including somatic node, its *relative energy consumptions* is defined as

4 $$\begin{aligned} E=\frac{F_{D}}{F_S\cdot (N-1)}, \end{aligned}$$

where $F_{D}$ and $F_S$ respectively represent the spiking number of dendrites and soma respectively within a time window considered. We consider here the same time window as in the calculation of dynamic range unless otherwise stated. In contrast to the dynamic range, the relative energy consumption *E* is associated with stimulus intensity *h*. Note that for sufficiently small *h*, relative energy consumption is small and fluctuate with a large variation, and for sufficiently large *h*, relative energy consumption saturate to $E=1$, as seen in Fig 7. Focusing on intermediate values of *h*, we take the average of relative energy consumption over intermediate values of *h* as a quantity to characterize the overall energy consumption; more precisely, we define the *average of relative energy consumption* as

5 $$\begin{aligned} E^*=\frac{\int _{10^1}^{10^3} E(h) dh}{10^3-10^1}. \end{aligned}$$

To investigate the efficiency of neuron computation among branches, we also compute the dynamic range of individual dendrites as well as the whole dentritic tree. We quantify the computational efficiency of branching by *average dynamic range ratio* *R*, defined as a relative ratio of average dynamic range among individual branches to that of the whole dendritic tree; more explicitly,

6 $$\begin{aligned} R=\frac{\sum _{i=1}^K{d_i}}{K\cdot D} \end{aligned}$$

where $d_i$ represents the dynamic range of the *i*-th subtree ($i=1,2,\cdots K$) (We remark here that in the calculation of $d_i$ soma is considered to be only connected to the *i*-th subtree, while in the calculation of *D* soma can receive activation from all branches in the tree). For single-branch dendrites $R=1$, while for multiple-branch dendrites $R\le 1$. A lower ratio *R* indicates the dynamic range of a dendrite *D* is much larger than the average dynamic range among subtrees $\sum _{i=1}^K d_i/K$, which suggests an effectively larger magnification of the stimulus range of afferent rate that dendrites recognize due to multiple branches.

## Data Availability

The data that support the findings of this study are available from the corresponding author on request.
